# Dr. Allen’s Letter

**Published:** 1852-04

**Authors:** 


### DR. ALLEN'S LETTER.

We give an extract from Dr. Allen's letter to the editors of the Recorder, which gives some good practical information, and imparts some of the principles on which his improvement depend.—Ed.

"But as the dental profession will derive no benefit from personal altercations, I wish to dwell more particularly upon the fundamental principles which serve as a basis for my improvement in artificial gums. With reference to recipes for enamels, I know of none that possess the requisite properties for uniting mineral teeth and metalic plates to each other, in such a manner as to form continuous gums, upon artificial dentures, without either cracking, scaling, or warping the plates upon which they are flowed, unless both sides are covered, which is found to be inadmissible for dental purposes. These are important principles, which must be observed, in order to form a continuous gum without encountering the difficulties above named, as the various efforts which have been made to attain this end, and have always proved abortive, clearly shows. In the first place, there should be a strong chemical affinity between the component parts of the compound, the teeth, and the plate upon which it is to be fused, in order that a perfect union of the whole should be formed. Among the various metals with which we are most familiar, we find that the linear dilatation of platinum by heat, when raised to 212° according to Borda, is 1 part in 1167, which is the least of any of the metals. Palladium, according to Wollaston, 1 in 1000, Antimony, 1 in 123; Cast Iron Bar, 1 in 901; Steel Bar, 1 in 862; Wrought Iron, 1 in 819; Bismith, 1 in 718; Gold, 1 in 713; Copper, 1 in 584; Brass, 1 in 548; Silver, 1 in 505; Tin, 1 in 438; Lead, 1 in 345; Zinc, 1 in 339.

Among the minerals we find that the same degree of temperature will produce a linear expansion. Marble, from St. Beat, 1 in 2391; Stone from St. Pernon, 1 in 2304; Stone from St. Leu, 1 in 1541. The clay which is obtained in Dorsetshire and Devonshire, 1 in 2123. In Wedgewood's wares, a large proportion of this material is used. Cornish stone, 1 in 1521; English flint glass, 1 in 1248; French plate, 1 in 1147; Sulphate of Barytes, 1 in 1204; Sulphate of Strontites, 1 in 1128; Asbestos, 1 in 1769; pure Silex, 1 in 1662.

From the metals above named, I select platina as the most suitable for plates for my own style of work, because the linear dilatation and contraction of this metal when exposed to extreme temperature of heat and cold, is less than in any other metal. The mineral compound to be employed in forming a continuous gum upon artificial dentures, should possess the same degree of resisting power as the plate upon which it is to be fused, otherwise the contractility of the mineral in cooling after fusion, (if greater than that of the plate) would either cause a crackling of the cement, or wrapping of the plate, which in either case would be highly detrimental. The same result would follow if the linear expansion, or contraction of the metal plate was greater or less than that of the mineral. Hence the importance of having the same degree of dilatation and contractility in the metal, and the mineral. Therefore if substances be used as fluxes, etc., for uniting the minerals which contract much, other substances more refractory in their nature should be added to the compound, such for instance as Strontia, Devonshire clay, or Wedgewood, Asbestos or stone from St. Pernon or St. Leu. These substances duly proportioned and properly prepared may be employed to great advantage in my silicious compound, which I employ in the construction of continuous artificial gums, as exemplified in my new style of work."

